# Ozymandias

**DOI:** 10.14797/mdcvj.697

**Published:** 2021-09-24

**Authors:** James B. Young

**Affiliations:** 1 Lerner College of Medicine of Case Western Reserve University, Cleveland, Ohio, US

## Abstract

I met a traveler from an antique land
Who said: Two vast and trunkless legs of stone
Stand in the desert….Near them, on the sand,
Half sunk, a visage lies, whose frown,
And wrinkled lip, and sneer of cold command,
Tell that its sculptor well those passions read
Which yet survive, stamped on these lifeless things,
The hand that mocked them, and heart that fed;
And on the pedestal these words appear:
“My name is Ozymandias, King of Kings;
Look on my works, ye Mighty, and despair!”
Nothing beside remains. Round the decay
Of that colossal wreck, boundless and bare
The lone and level sands stretch far away.

Percy Bysshe Shelley
*Rosalind and Helen, A Modern Eclogue; With Other Poems*
London: Hollinger. p. 72 (1876)

This poem is in the public domain.

## Tempus Fugit

Percy Bysshe Shelley (1792–1822) and his second wife, Mary Shelley (married 1816 and author of *Frankenstein*, 1818), are known to many as classic English Romantic period authors. Percy was a major player in that literary scene with shorter works such as *Ozymandias* (1818), *Ode to the West Wind* (1819), and *To a Skylark* (1820), all well-known and studied still in many introductory literature courses. He also was known for his longer works that included allegorical tales such as *Adonais* (1821) and *Prometheus Unbound* (1820), but arguably, he is best remembered for the sonnet *Ozymandias*. Shelley tragically died in a boating accident at age 29. Although not renowned while alive, he gained fame posthumously and influenced other great poets such as Browning and Yeats.

*Ozymandias* grew from a conversation Shelley had with an acquaintance in 1817 while living in Italy. In the aftermath of Napoleon’s Egyptian military victories (1798), many archeologic wonders were found, prompting consideration of the very colorful and remote pharaonic past. Those days seemed indestructible, but in Shelley’s time, the grand and storied empire was long gone and left in sandy ruin. In an essay dubbing *Ozymandias* “a poem to outlast empires,”^1^ David Mikics noted that Shelley was aware of Diodorus Siculus, a Roman-era historian whose writings describe the Ozymandias statue. Diodorus noted that perhaps Ozymandias was the Biblical Exodus pharaoh, Rameses II, and suggested that the inscription on the statue (the largest in Egypt at the time) was “King of Kings Ozymandias am I. If any want to know how great I am and where I lie, let him outdo me in my work.” In Shelley’s poem, Diodorus becomes the “traveler from an antique land” who might have seen the statue. Shelley did not. He was reacting to an announcement by the British Museum that the massive torso and head portion of a colossal Ramesses II statue from 13th century BCE, discovered in 1816 by an Italian explorer in Thebes, was soon to arrive in England. Shelley’s poem ended up being published before the statue arrived, and he died shortly thereafter.

It is important to recall the theme of *Ozymandias*, written in sonnet style iambic pentameter with an atypical rhyming scheme. Evolution is history, and history is constantly evolving. Only the pace varies, such as with scientific discovery, and only those things we know about and understand can we know. Philosophers have said that one can dip their toe into a moving stream only once, because the stream is constantly changing. Heraclitus, the weeping Greek philosopher seen crying at the foot of the stairs in the iconic Vatican Library *School of Athens* painting (Raphael 1509–1511), has been said to weep in angst because of life’s constant change and the folly of mankind. And so it is that the story of *Ozymandias* and the colossus statue is but a metaphor for change, evolution, and the fate and folly of man. It makes me think of the great natural feats blessing our earth, particularly the evolution of our geologically older mountain ranges (the Adirondack, Allegheny, and Smoky Mountains) and our newer ones (the Rocky Mountains and Sierra Nevadas); the massive glaciers carving out extraordinary visages such as California’s Yosemite Valley, Kings Canyon, Devils Postpile, and the Minarets as a few examples.

Thinking of what glaciers can do brings up the issue of global warming as another example of change and evolution. Climate change can’t be argued. The ice age left Kansas long ago. Ohio’s “Kettle” lakes and the scarred valleys that I see with my wife during our weekend hikes are another testament of the past. Perhaps the speed of change can be debated, as can the influence of the way we live (Heraclitus’ “folly of man”). And of course, there are many more examples of change and evolution in our profession of medicine. Just think, as we are coming out of pandemic lockdown, about the evolution of coronavirus. Inevitable and fascinating.

*Ozymandias* is a great poem to read, study, and ponder. It is a true classic published over two centuries ago and withstanding the test of time. It is interesting to vision implications of the sands of Egypt slowly blowing over an empire (again, an allegory) and changing things forever, particularly the hubris of pharaohs and the very life lived in those times. “I met a traveler from an antique land…” and it made me think of all that is good with change and evolution, but that human sensibility must not be sundered if we expect to beat back blowing sands.

Enjoy *Ozymandias*—it is best when read aloud.

## References

[1] Mikics D. Ozymandias. In: Burt S, Mikics, D. The Art of the Sonnet. Cambridge, Mass.: The Belknap Press of Harvard University Press; 2010. p.125–129.

